# Healthy women with severe early life trauma show altered neural facilitation of emotion inhibition under acute stress

**DOI:** 10.1017/S0033291719002198

**Published:** 2020-09

**Authors:** Sabrina Golde, Katja Wingenfeld, Antje Riepenhausen, Nina Schröter, Juliane Fleischer, Jens Prüssner, Simone Grimm, Yan Fan, Julian Hellmann-Regen, Anne Beck, Stefan M. Gold, Christian Otte

**Affiliations:** 1 Charité – Universitätsmedizin Berlin, Corporate Member of Freie Universität Berlin, Humboldt-Universität zu Berlin, and Berlin Institute of Health, Klinik für Psychiatrie und Psychotherapie, Campus Benjamin Franklin, Berlin, Germany; 2 Charité – Universitätsmedizin Berlin, Corporate Member of Freie Universität Berlin, Humboldt-Universität zu Berlin, and Berlin Institute of Health, Klinik für Psychiatrie und Psychotherapie, Campus Charité Mitte, Berlin, Germany; 3 Department of Psychology, University of Konstanz, Konstanz, Baden-Württemberg, Germany; 4 Department of Psychiatry, Psychotherapy and Psychosomatics, Hospital of Psychiatry, University of Zurich, Zurich, Switzerland; 5 MSB Medical School Berlin, Berlin, Germany; 6 Institut für Neuroimmunologie und Multiple Sklerose (INIMS), Zentrum für Molekulare Neurobiologie, Hamburg, Germany

**Keywords:** Acute psychosocial stress, anterior insula, early life trauma, emotion inhibition, fMRI, inferior frontal gyrus

## Abstract

**Background:**

Across psychopathologies, trauma-exposed individuals suffer from difficulties in inhibiting emotions and regulating attention. In trauma-exposed individuals without psychopathology, only subtle alterations of neural activity involved in regulating emotions have been reported. It remains unclear how these neural systems react to demanding environments, when acute (non-traumatic but ordinary) stress serves to perturbate the system. Moreover, associations with subthreshold clinical symptoms are poorly understood.

**Methods:**

The present fMRI study investigated response inhibition of emotional faces before and after psychosocial stress situations. Specifically, it compared 25 women (mean age 31.5 ± 9.7 years) who had suffered severe early life trauma but who did not have a history of or current psychiatric disorder, with 25 age- and education-matched trauma-naïve women.

**Results:**

Under stress, response inhibition related to fearful faces was reduced in both groups. Compared to controls, trauma-exposed women showed decreased left inferior frontal gyrus (IFG) activation under stress when inhibiting responses to fearful faces, while activation of the right anterior insula was slightly increased. Also, groups differed in brain–behaviour correlations. Whereas stress-induced false alarm rates on fearful stimuli negatively correlated with stress-induced IFG signal in controls, in trauma-exposed participants, they positively correlated with stress-induced insula activation.

**Conclusion:**

Neural facilitation of emotion inhibition during stress appears to be altered in trauma-exposed women, even without a history of or current psychopathology. Decreased activation of the IFG in concert with heightened bottom-up salience of fear related cues may increase vulnerability to stress-related diseases.

## Introduction

Exposure to trauma is a significant risk factor for psychopathology (Nemeroff, 2004). However, some individuals exposed to severe traumatic events do not develop full-blown psychiatric disorders. There are growing indications that these trauma-exposed individuals show neural alterations, even in the absence of clinical symptoms (Stark *et al*., 2015; Teicher and Samson, 2016). Tasks requiring the regulation of emotions and impulses seem to be particularly affected. Concurrently, in trauma-exposed psychiatric patients, significant impairments in these domains are observed, across psychopathologies. These deficiencies are believed to be responsible for hallmark trauma-associated psychiatric symptoms such as hyperarousal in post-traumatic stress disorder (PTSD) (Shalev *et al*., 2017) and depression (Goldsmith *et al*., 2013) or impulsivity in borderline personality disorder (van Zutphen *et al*., 2015).

Previously, studies have extracted distinct neural dysregulations associated with PTSD by comparing PTSD patients to trauma-exposed controls (Fani *et al*., 2012*a*, 2012*b*). More recently, there has been an increasing focus on trauma-exposed participants without clinical symptoms. Despite some earlier reports of decreased inhibitory performance associated with trauma (Aupperle *et al*., 2012), trauma-exposed participants without current psychopathology frequently do not suffer from significant behavioural impairments (Falconer *et al*., 2008; Covey *et al*., 2013; Quidé *et al*., 2017; Melara *et al*., 2018). However, studies of neural underpinnings paint a complex picture. For example, a recent fMRI-based study by Quidé *et al*. (2017) examined non-affective response inhibition in patients with psychotic disorders and healthy controls. While not finding any effects of psychosis, the authors found altered left inferior frontal gyrus (IFG) functioning in a subgroup of participants that was exposed to childhood trauma. Furthermore, Melara *et al*. (2018) compared neural correlates of distractor inhibition between PTSD patients, trauma-exposed participants without PTSD and trauma-naïve healthy controls by means of EEG. Results were generally highly similar for both non-PTSD groups. However, in trauma-exposed without PTSD only, the ventromedial prefrontal cortex was associated with inhibitory control. Thus, these participants may require more active inhibition when processing emotional stimuli than trauma-naïve participants. An EEG-based study conducted by Covey *et al*. (2013) compared response inhibition of trauma-exposed policemen without the current psychiatric disorder to trauma-naïve civilian controls. Here, greater P3 amplitude, suggesting greater arousal, was found in police men compared to controls. An earlier fMRI study by Falconer *et al*. (2008), however, examining response inhibition did not find any neural differences between healthy trauma-exposed and trauma-naïve controls.

Overall, previous results are highly heterogeneous. They may point to subtle alterations that remain difficult to detect but can figure prominently. In fact, it has been suggested that differences in inhibitory functioning increase vulnerability to subsequent psychopathology (Teicher and Samson, 2016). Alternatively, they may mark psychiatric resilience, either as a pre-existing factor or as a product of early stress experiences. Notably, previous studies have not controlled for a lifetime diagnosis of any psychiatric disorder. Also, associations with clinical symptomatology in these trauma groups are poorly understood.

Importantly, differences may amplify in more demanding environments that induce everyday life stress. Subtle deficits in emotion regulation and inhibition may not significantly influence daily functioning until acute, ordinary and non-traumatic stress perturbates the system. Stress strengthens threat detection and rapid reactions but at the same time, it decreases the ability to inhibit automatic reactions (Starcke and Brand, 2012; Shields *et al*., 2016). Furthermore, the sensitivity of neural emotion regulation processes to psychological everyday stress might be particularly relevant for the aetiology of trauma-related disorders (Nemeroff, 2004).

Several candidate brain areas are known to play an important role in top-down inhibition of emotions. The ventrolateral prefrontal cortex including the IFG represent the core areas facilitating response inhibition and top-down control of emotion (Kohn *et al*., 2014). Also, the bilateral middle frontal gyrus, as well as the anterior cingulate and medial prefrontal cortex, have consistently been associated with emotion inhibition (Cromheeke and Mueller, 2014; Kohn *et al*., 2014). Moreover, the anterior insula fulfils a central role in emotion regulation processes by marking events as salient and coordinating the involvement of different, large-scale neural networks (Menon and Uddin, 2010; Menon, 2011). Previous studies have further suggested that trauma-related psychopathology is associated with altered neural responses during emotion processing and inhibition in the lateral and medial prefrontal cortex as well as anterior insula (van Zutphen *et al*., 2015; Aupperle *et al*., 2016). Some studies in trauma-exposed participants without current clinical symptoms have pointed to only slight anomalies in overlapping areas (Stark *et al*., 2015).

However, it is unknown how these neural systems in trauma-exposed individuals react to demanding environments and challenging circumstances. In this study, we therefore examined the effect of acute ordinary, non-traumatic stress on subsequent emotion inhibition in healthy women who had experienced multiple severe sexual or physical traumatic events before the age of 18, but never developed any psychiatric disorder (neither on DSM Axis I nor II; T+) and matched trauma-naïve healthy controls (T−).

We expected no significant differences in behavioural performance between trauma-exposed and trauma-naïve individuals, and neural differences to be subtle under baseline conditions. However, under stress, we hypothesized that trauma-exposed participants would, compared to trauma-naïve participants, show decreased top-down control, particularly in the IFG, and increased activation in areas of emotion and salience processing (Stark *et al*., 2015; Homberg *et al*., 2017). In addition, we assessed the exploratory hypothesis that healthy trauma-exposed participants might show different (compensatory) mechanisms to facilitate emotion inhibition.

## Methods

### Participants

Twenty-five healthy women who experienced multiple severe sexual or physical traumatic events (T+) were recruited. The inclusion criterion for this group was a minimum of three traumatic events (sexual or physical) before the age of 18 with neither a history of nor current psychiatric disorders. We conducted a thorough pre-screening via phone (duration approximately 40 min) to pre-assess the authenticity of the trauma criterion. In addition, 25 matched healthy control participants without a history of trauma (T−) participated. All were recruited via public advertisements. Participants underwent a detailed diagnostic interview by a trained clinical psychologist to assess inclusion and exclusion criteria. For both groups, psychiatric disorders (lifetime and current) were assessed by the Structured Clinical Interviews for DSM-IV Axis I and II (SCID I and II; Wittchen *et al*., 1997). The inclusion criterion of severe early life trauma (sexual or physical) was further assessed using the German version of the Early Trauma Inventory (ETI; Bremner *et al*., 2000; Wingenfeld *et al*., 2011), a 56-item semi-structured interview for the assessment of physical, emotional and sexual abuse as well as general traumatic experience. The interview served to obtain in-depth information as well as specific (e.g. onsets, offsets, perpetrators, etc.) information about the events and to assess authenticity of the report. In order to collect an interviewer-independent measure and to increase comparability with previous studies, participants additionally completed the Childhood Trauma Questionnaire (CTQ; Bernstein *et al*., 2003). All participants of the trauma group also fulfilled Criterion A for PTSD of the DSM-V. This criterion requires the person having been exposed to ‘death, threatened death, actual or threatened serious injury, or actual or threatened sexual violence’ (American Psychiatric Association, 2013).

Criteria for exclusion were (1) lifetime diagnosis of a psychiatric disorder as assessed by the Structured Clinical Interviews for DSM-IV Axis I and II (SCID I and II; Wittchen *et al*., 1997), (2) adverse health conditions affecting the central nervous or endocrine system function, (3) non-removable ferromagnetic material, (4) auto-immune and infectious diseases, (5) hypertension, (6) a transcontinental flight within the last 4 weeks, (7) excessive physical exercise of more than 10 h a week and (8) left-handedness. The study was conducted in accordance with the latest version of the Declaration of Helsinki and received approval by the local ethics committee (protocol number EA4/104/13). All participants provided written informed consent and were financially reimbursed.

Two T+ and two T− participants had to be excluded due to excessive head movement (more than 3 mm translation or 3° rotation). These participants were hence excluded from all analyses.

### Study design and procedure

The study consisted of a diagnostic and an experimental session that took place on different days within a 2-week period. During the diagnostic session, inclusion and exclusion criteria were assessed and the following self-report questionnaires were obtained: Beck Depression Inventory II (BDI II; Beck *et al*., 1996), the Spielberger State-Trait Anxiety Inventory (STAI-T; Spielberger *et al*., 1983), the Posttraumatic Diagnostic Scale (PDS; Foa *et al*., 1997), the Perceived Stress Scale (PSS; Cohen *et al*., 1983) as well as the Stress Reactivity Scale (SRS; Schulz *et al*., 2005).

**Fig. 1.**
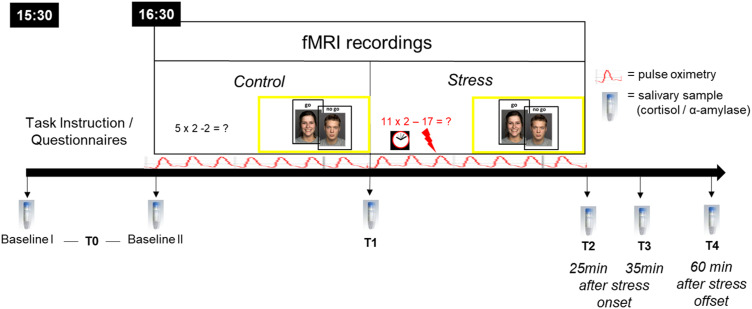
Experimental procedure. All participants arrived at 15:30 h at the laboratory to control for circadian rhythmicity of cortisol release, fMRI testing began at 16:30 h. During fMRI, participants completed two runs of an emotional go-nogo (eGNG) paradigm (yellow boxes), one in the control condition and one after psychosocial stress induction. Six salivary samples were taken over the course of the experimental session. Baseline I (upon arrival) and II (before scanning, 45 min later) were averaged to a single baseline value (T0) to reduce situational influences on baseline measures. The T1 sample was taken inside the scanner, in-between control and stress condition, approximately 20–25 min after Baseline II. T2 = 25 min after stress onset, T3 = 35 min after stress onset, T4 = 60 min after stress offset. Pulse oximetry was used to measure heart rate over the course of the fMRI session.

During the experimental session, participants completed two runs of an emotional go-nogo (eGNG) paradigm during fMRI, one before and one after psychosocial stress induction by an adapted version of the Montreal Imaging Stress Task (MIST; Dedovic *et al*., 2005; Pruessner *et al*., 2008). The experimental procedure is depicted in Fig. 1, and the fMRI paradigm is described in more detail below.

#### eGNG paradigm

We used a modified version of an eGNG paradigm previously employed by Hare *et al*. (2008). Subjects completed two runs (one in the control and one in the stress condition), comprised of four blocks each. Each block consisted of 30 go-trials and 10 nogo-trials. During go-trials, a target facial expression was presented, and subjects were asked to respond as fast as possible by pressing a button. During nogo-trials, non-target facial expressions were presented and subjects were asked to avoid any button press (inhibition trials). Two blocks used emotional target facial expressions (fearful or happy, respectively) in the presence of neutral non-targets, and two blocks employed neutral target faces, one in the presence of fearful non-targets and one in the presence of happy ones. Together with the participants' response, this task design provides for the analysis of four different trial types: correct go-trials (hits), correct nogo-trials (inhibition trials), incorrect nogo-trials (false alarms) and incorrect go-trials (misses).

At the beginning of each block, written instructions were displayed telling the subject to respond to the target facial expression as fast as possible by pressing with the right thumb and not to press any button for non-target facial expressions. In addition, the current task was verbally explained to the participants by the experimenter over the microphone. See Supplementary materials M1 and methods for details on stimuli selection, presentation and timing.

#### Stress induction and response measures

For psychosocial stress induction, we employed an adapted version of the MIST (Dedovic *et al*., 2005; Pruessner *et al*., 2008). See Supplementary materials and methods M2 for details. In short, during the control condition, easy arithmetic questions were presented. Stress induction then consisted of difficult arithmetic questions under time pressure in addition to negative social feedback.

To evaluate subjectively experienced stress, we asked participants to rate their stress and strain level on a 10-point scale during the control and stress condition after the experiment. Heart rate was recorded using the integrated photoplethysmograpth of the Siemens Physiological Monitoring Unit under the left index finger. As a manipulation check of physiological stress induction, six saliva samples were collected, the first two were averaged as a single baseline value (T0) to reduce situational influences on baseline measures (see Fig. 1). Details on peak detection and biochemical analysis of saliva samples are provided in the Supplementary materials and methods M3 and M4.

#### fMRI first-level model

Please refer to Supplementary materials and methods M5 for details on acquisition parameters and pre-processing. Effects were estimated using an event-related general linear model convolving each trial with a haemodynamic response function. A fixed-effect model was performed to create images of parameter estimates. We modelled four different emotion conditions, corresponding to four blocks of trials. These were blocks with (1) fearful nogo faces–neutral go faces, (2) happy nogo faces–neutral go faces, (3) neutral nogo faces–fearful go faces, (4) neutral nogo faces–happy go faces. For each emotion condition, we also modelled the control and stress condition separately. Within each control/stress condition and each emotion condition in turn, we modelled four different trial types: correct go trials, correct nogo trials, false alarms (incorrect go), misses (incorrect nogo). Thus there were 4 (emotion condition) × 4 (trial type) × 2 (stress *v.* control condition) regressors. Regressors containing correct nogo trials were the regressors of interest. Please refer to online Supplementary Table S1 for a complete list of regressors and belonging trials. Additionally, realignment parameters were included as additional regressors in the model. Individual *t*-contrast maps of (stress>control) for all correct inhibition (nogo) trials of all emotion conditions were computed.

### Statistical group analysis

#### Sample characteristics and stress induction measure

All non-imaging data related analyses were carried out using IBM SPSS Statistics 22 for Windows (SPSS Inc., Chicago, IL, USA). Demographic and clinical data were analysed with two sample *t* tests or χ^2^ tests. Salivary data was winsorized (95^th^ percentile) and log-transformed. Subjective stress, mean heart rate, cortisol and *α*-amylase values were analysed with a mixed design analysis of variance (ANOVA), Greenhouse-Geisser correction was used when appropriate. Post-hoc *t* tests were Bonferroni corrected.

#### eGNG paradigm

*Behavioural*. We explored stress-induced failures of response inhibition as indexed by false alarm rate (FAR) on inhibition (nogo) trials in the stress *v.* control condition (cf. Wager *et al*., 2005). First, the effect of stress on FAR in all four nogo emotion conditions: fearful, happy, neutral (fearful go), neutral (happy go) was assessed by Wilcoxon signed-rank testing. Second, the stress-induced increase ΔFAR was calculated by subtracting the control from the stress runs and Mann–Whitney *U* testing was employed to test group differences in ΔFAR. The Bonferroni method was used to correct for multiple testing.

*Imaging* (*group level*). On the group level, individual *t*-contrast images (stress>control) from first level were entered into a flexible factorial ANOVA with group (T+ *v*. T−), emotion condition (fearful nogo, happy nogo, neutral nogo_fear−go_, neutral nogo_happy−go_) and subject as factors. We used probabilistic threshold-free cluster enhancement (pTFCE) (Spisák *et al*., 2019) in addition to whole brain peak-level (voxel-wise) FDR correction with *p* < 0.05 and a minimum cluster size threshold of *k* > 30.

## Results

### Stress induction

Demographic and clinical data are shown in Table 1.

**Table 1.** Sample characteristics

	T+ (M ± SD)	T− (M ± SD)	Statistics
Age (years)	31.52 ± 9.71	31.22 ± 10.42	*p* = 0.919
Education (years)	11.57 ± 0.99	11.61 ± 0.89	*p* = 0.876
Intake of OC (no/yes)	20/3	16/7	*p* = 0.153
Cycle phase if no intake of OC (follicular/luteal/postmeno)	5/12/2	3/11/2	*p* = 0.543
Smoker (yes/no)	6/17	5/18	*p* = 0.730
Coffee per day (cups)	1.32 ± 1.22	1.73 ± 1.74	*p* = 0.370
BDI II	8.23 ± 6.19	2.91 ± 4.04	***p* = 0.001**
STAI-T	38.30 ± 9.83	33.26 ± 6.71	***p* = 0.048**
PDS	10,87 ± 10.68	0.96 ± 2.33	***p* < 0.001**
PSS	16.44 ± 6.25	14.35 ± 4.64	*p* = 0.205
SRS	56.64 ± 7.93	56.83 ± 6.57	*p* = 0.201
CTQ sum score	66.57 ± 16.43	28.82 ± 3.50	***p* < 0.001**
Emotional abuse	16.96 ± 6.09	5.74 ± 1.18	***p* < 0.001**
Physical abuse	11.78 ± 4.82	5.04 ± 0.21	***p* < 0.001**
Sexual abuse	10.30 ± 6.07	5.00 ± 0.00	***p* < 0.001**
Emotional neglect	17.00 ± 5.48	7.43 ± 2.45	***p* < 0.001**
Sexual neglect	10.52 ± 4.76	5.57 ± 1.31	***p* < 0.001**
ETI sum score	490.87 ± 342.40	14.52 ± 13.25	***p* < 0.001**
General trauma	91.17 ± 73.45	8.78 ± 10.31	***p* < 0.001**
Physical abuse	100.48 ± 75.20	2.70 ± 4.63	***p* < 0.001**
Emotional abuse	271.61 ± 230.52	1.30 ± ± 4.07	***p* < 0.001**
Sexual abuse	27.61 ± 48.97	1.74 ± 3.62	***p* = 0.019**

T+, trauma-exposed participants; T−, trauma-naïve control participants; OC, oral contraceptives; BDI II, Beck Depression Inventory II; STAI-T, Spielberger Trait Anxiety Inventory; PDS, Posttraumatic Stress Diagnostic Scale; PSS, Perceives Stress Scale; SRS, Stress Reactivity Scale; CTQ, Child Trauma Questionnaire; ETI, Early Trauma Inventory.

Information on cycle phase was unavailable for one T+ due to uterine agenesis.

#### Subjective stress and heart rate increases

An ANOVA for subjective stress showed a main effect of stress indicating higher values in the stress compared to the control condition (*F*_1,42_ = 88.4, *p* < 0.001), but no main effect of group or group by stress interaction. For subjective strain, we also saw a significant main effect of strain (*N* = 44, *F*_1,42_ = 92.7, *p* < 0.001) and a significant condition by group interaction, indicating higher strain increase in T+ (*F*_1,42_ = 7.1, *p* = 0.042, see online Supplementary Fig. S1).

**Fig. 2.**
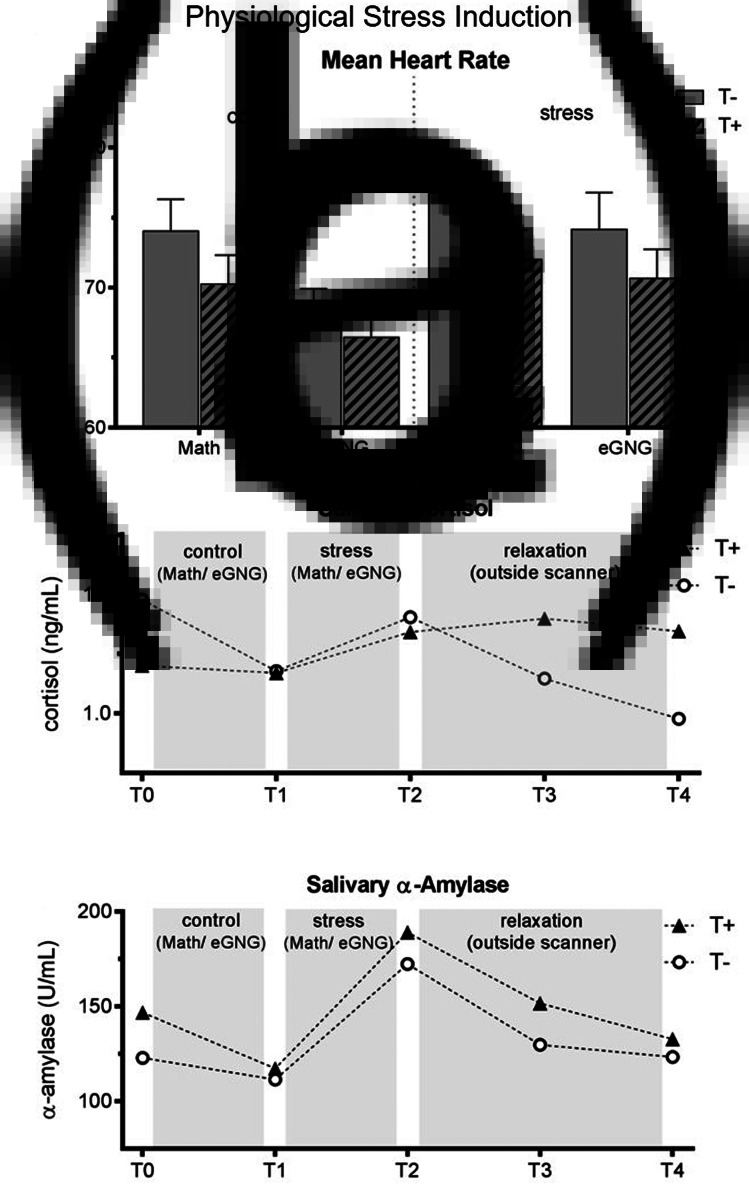
(*a*) Mean heart rate over the experiment. Heart rate data of eight participants (3 T+ and 5 T−) had to be excluded due to scanner and movement related artefacts. We computed a mixed ANOVA including condition (control *v*. stress) and task (MIST *v*. eGNG) as within-subject factors and group as a between-subject factor. A significant main effect of condition (*N* = 38, *F*_1,37_ = 13.8, *p* = 0.001) demonstrated elevated heart rate in the stress condition compared to control, while a main effect of task (*N* = 38, *F*_1,37_ = 61.8, *p* < 0.001) indicated higher mean heart rate during presentation of math questions than during the eGNG paradigm. There was no main effect of group and no significant interactions. (*b*) Raw salivary cortisol data. Before the analysis of salivary cortisol and salivary *α*-amylase levels, one participant (T−) had to be discarded due to food consumption at sampling time. For statistical analysis, data were winsorized and log-transformed. Two mixed ANOVAs including a within-subject factor time (5 measurement points) and a between-subject factor group were conducted. For cortisol, we found a main effect of time (*N* = 45, *F*_4,43_ = 4.8, *p* = 0.009), but no main effect of group or interaction effect. Post-hoc Bonferroni corrected paired samples *t* tests examining the increase during stress condition (T1 *v*. T2) and subsequent recovery (T2 *v*. T3) across all participants showed a significant cortisol increase during stress (T1 *v*. T2: *N* = 45, *t*_44_ = 2.4, *p* = 0.044 adjusted) and recovery afterwards (T2 *v*. T3: *N* = 45, *t*_44_ = 5.2, *p* = < 0.001 adjusted). (*c*) For *α*-amylase, there was a main effect of time (*N* = 45, *F* = _4172_ = 17.3, *p* < 0.001), no main effect of group, but a time by group interaction (*N* = 45, *F*_4172_ = 2.6, *p* = 0.037). To decode the interaction, we conducted Bonferroni corrected paired sample *t* tests between all consecutive time points (T1 *v.* T2, T2 *v.* T3, etc.) for both groups separately (four tests per group). This analysis revealed a marginally significant salivary *α*-amylase decrease from T0 to T1 in T+ participants (*N* = 23, *t*_22_ = 2.7, *p* = 0.056 adjusted) but not in T− participants (*p* > 0.999 adjusted), as well as large increases from T1 to T2 in both groups (T+ group: *N* = 23, *t*_22_ = 7.4, *p* < 0.001 adjusted, *d* = 1.6; T− group: *N* = 22, *t*_21_ = 3.8, *p* = 0.004 adjusted, *d* = 0.9). There were no significant differences in any other paired tests. Thus, the interaction was attributable to group differences in *α*-amylase changes from T0 to T1, resulting from higher *α*-amylase baseline levels (T0) in the T+ group (two-sample *t* test of T0 values: *N* = 45, *t*_44_ = 2. 2, *p* = 0.030). See online Supplementary Table S3 for all means and standard errors. T−, trauma-naïve control participants; T+, trauma-exposed participants; control, control condition; stress, stress condition; eGNG, emotional go-nogo paradigm; bpm, beats per minute.

Heart rate was significantly higher during the stress compared to the control condition as well as during the MIST compared to eGNG in both groups (see Fig. 2*a* and online Supplementary Table S2).

#### Salivary cortisol and *α*-amylase

Cortisol levels significantly increased during stress induction (T2 to T3) and significantly decreased afterwards (T3 to T4), there were no group differences or time by group interactions. The *α*-amylase levels also significantly increased during stress induction (T2 to T3) in both groups; in addition, the T+ group had significantly higher baseline values (T1; see Fig. 2*b*–*c*).

### Emotional response inhibition

#### Behavioural

**Fig. 3.**
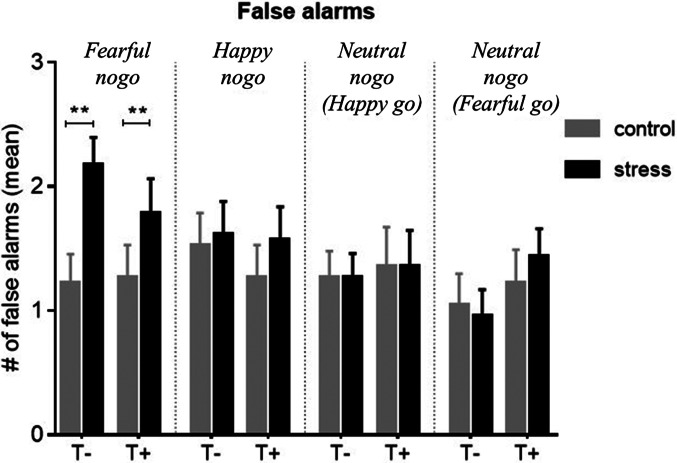
Mean false alarm rate (FAR) for non-target (nogo) trials of all emotional conditions, compared between control and stress conditions. There was a significant stress-induced increase in FAR on fearful non-targets in both groups (*p* < 0.001), but no group differences. Stress had no significant effect on FAR in any other emotion condition. Control, control condition; stress, stress condition; T+, trauma-exposed participants; T−, trauma-naïve control participants.

The effect of stress on FARs on nogo stimuli in the four emotion conditions was examined across all participants. Stress significantly increased FAR on fearful faces (Wilcoxon: *N* = 46, *Z* = −3.6, *p* *<* 0.001 adjusted, *r* = 0.5), but had neither an effect on happy ones (*N* = 46, *Z* = −1.1, *p* = 0.29 unadjusted) nor on one of the neutral stimuli conditions (*fearful go*: *N* = 46, *Z* = −0.3, *p* = 0.76 unadjusted; *happy go*: *N* = 46, *Z* = −0.04, *p* = 0.98 unadjusted) (Fig. 3). The stress-associated increase in false alarms ΔFAR did not significantly differ between the groups for any emotion condition [Mann–Whitney *U* tests: *N* = 46 for all, *Z*_happy_ = −0.9, *p* = 0.39, *Z*_fear_ = −0.8, *p* = 0.46, *Z*_neut(happy−go)_ = −0.2, *p* = 0.84, *Z*_neut(fear−go)_ = −0.8, *p* = 0.46]. Thus, stress decreased inhibition of fearful nogo stimuli to a similar degree in both groups but had no effect on happy or neutral ones. Statistics on go stimuli are provided in online Supplementary Table S4.

#### Imaging

**Fig. 4.**
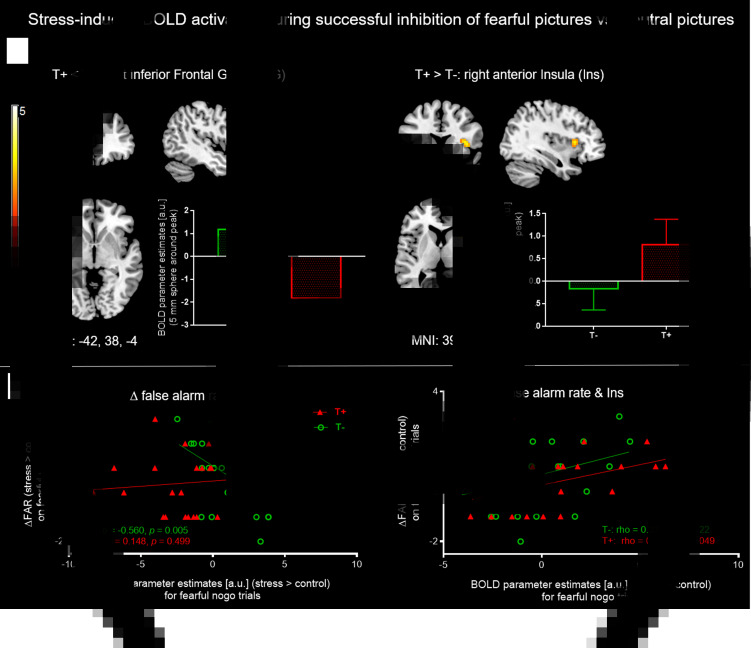
(*a*) Individual *t*-maps of stress-induced BOLD increases (stress > control) for non-target (i.e. nogo) trials of all four emotion conditions were entered into a flexible factorial ANOVA and for both groups, fearful nogo-pictures were compared to neutral nogo ones [|fearful-nogo – neutral-nogo| × group]. The T− group showed higher stress-associated left IFG activation (left), whereas T+ participants showed marginally higher stress-associated right anterior insula activation. Bar graphs depict mean BOLD parameter estimates from a 5 mm sphere around the clusters' peak voxel. For IFG, results are pTFCE and FDR peak-corrected (*p* < 0.05) for the whole brain, minimum cluster size *k* > 30 voxels. For anterior insula, results are uncorrected, *p* < 0.001, *k* > 30. (*b*) Left: Significant negative Spearman correlation between ΔFAR (increase in FAR on fearful nogo trials from control to stress condition) and stress induced left IFG activation during fearful nogo-trials (i.e. stress > control) in T− controls but not T+ participants. Right: Significant positive Spearman correlation between ΔFAR and stress-induced right insula activation during fearful nogo-trials in the T+ but not the T− group. IFG, inferior frontal gyrus; FAR, false alarm rate; T−, trauma-naïve control participants; T+, trauma-exposed participants; control, control condition; stress, stress condition; a.u., arbitrary unit.

Results for neural correlates during stress induction (MIST) can be found in online Supplementary Tables S5 and S6. As stress only affected response inhibition of fearful nogo stimuli, we focused on exploring neural foundations of this stress effect on fearful stimuli in both groups. The analysis was hence on average based on 26.3 correct nogo trials for baseline and another 25.5 correct nogo trials for the stress condition. The principal contrast compared fearful nogo to neutral nogo faces and tested for an interaction effect with the group factor, i.e. [|fearful nogo–neutral nogo| × group]. This analysis revealed a significant interaction in the left IFG, indicating that T+ individuals showed blunted left IFG activation under stress compared to T− participants (MNI: −42, 38, −4, *t* = 5.33, *p* = 0.010 pTFCE and peak-level FDR corrected, 355 voxels). The reverse interaction contrast testing for increased neural activation under stress in the T+ group compared to the T− group hinted at a higher stress-induced activation in the T+ group in the right anterior insula (aIns) compared to the T− group, which however did not survive whole brain pTFCE and FDR correction (MNI: 39, 17, 5, *t* = 3.65, *p* = 0.5 pTFCE and peak-level FDR corrected, *p* *<* 0.001 *uncorrected*, 40 voxels) (Fig. 4*a*). Additionally, an analysis was run comparing the subgroup of neutral nogo trials that were part of fearful go-blocks with fearful nogo trials [|fearful nogo–neutral nogo_fearful−go_| × group]. Here, we found that the anterior insula cluster was slightly larger (IFG: cluster: MNI: −39, 38, −1, *t* = 5.51 *p* = 0.037 TFCE and peak-level FDR corrected, 305 voxels; insula: MNI: 33, 14, 8, *t* = 4.8, *p* = 0.1 TFCE and peak-level FDR corrected, *p* < 0.001 uncorrected, 57 voxels). To assess whether our findings were lateralized, we lowered the threshold to *p* < 0.001 uncorrected, *k* > 5 voxels, but did not see the respective bilateral activation difference (i.e. in the right IFG or left anterior insula). Next, we extracted mean BOLD parameter estimates of the left IFG and right anterior insula (5 mm sphere around peak voxel) from individual contrast images showing stress *v.* control during fearful nogo trials and analysed associations with a stress-induced increase in FAR. A sphere around the peak has been shown to be a reliable and sensible single-value ROI and was therefore chosen instead of the whole cluster (Tong *et al*., 2016). The Spearman correlation coefficient was used to test for bivariate correlations as a stress-induced increase in FAR values deviated significantly from a normal distribution.

For the left IFG, activation increase under stress in the T− group correlated negatively with a stress-induced increase in FAR (*N* = 23, Spearman *ρ* = −0.6, *p* *<* 0.01), which was not observed in the T+ group (*N* = 23, Spearman *ρ* = 0.1, *p* = 0.50). This indicates that in T− participants, higher left IFG activation during stress tends to amount to better response inhibition of fearful faces under stress (Fig. 4*b*, left).

For the right anterior insula, we observed that stress-induced activation in the T+ group was positively related to a stress-induced increase in FAR (*N* = 23, Spearman *ρ* = 0.4, *p* = 0.049), but not in the T− group (*N* = 23, Spearman *ρ* = 0.3, *p* = 0.12). Thus, a higher activation of the right anterior insula in T+ participants is associated with a higher increase in FAR on fearful nogo faces (Fig. 4*b*, right).

Furthermore, we analysed associations of stress-induced neural activation with psychiatric anomalies (posttraumatic symptom severity by PDS, trait anxiety by STAI-trait, depressive symptoms by BDI-II). Left IFG activation for the whole sample correlated negatively with PDS total score (*N* = 46, Spearman *ρ* = −0.568, *p* < 0.001), as well as with all PDS subscales, see online Supplementary Fig. S2. There was also a significant negative correlation of left IFG activation with STAI-trait (*N* = 46, Spearman *ρ* = −0.370, *p* = 0.011), but none with BDI-II. There was no significant correlation between stress-induced insula activation and psychiatric symptoms.

## Discussion

We investigated response inhibition in an eGNG paradigm using emotional face stimuli. We compared women who had experienced severe and multiple traumatic events before the age of 18 (T+) but have not (yet) developed any psychiatric disorder, with trauma-naïve control subjects (T−). In both groups, non-traumatic stress significantly impaired response inhibition of fearful facial expressions but did not affect inhibition of happy or neutral stimuli. When inhibiting responses to fearful faces under stress, T+ participants demonstrated a decreased brain signal in the left IFG but a marginally increased signal in the right anterior insula compared to T− controls. In T− only, IFG activation was linked to a lower rate of stress-induced false alarms, whereas in T+, insula activation was linked to a higher number of false alarms. To our knowledge, this is the first study demonstrating that trauma, in the absence of lifetime psychiatric disorder, affects neural emotion inhibition under acute stress.

While the IFG represents a central area for the top-down cognitive control of emotions (Cromheeke and Mueller, 2014), the insula is seen as the neural locus for bottom-up salience detection and interoception (Menon and Uddin, 2010). It has been suggested that stress temporarily lowers top-down control in exchange for a reallocation of resources to salience detection (Homberg *et al*., 2017). Our results show blunted IFG activation under stress when inhibiting responses to fearful faces in T+ participants compared to T− controls. Additionally, we found a trend towards increased response from the anterior insula in T+ participants under stress. Taken together, this pattern points to more pronounced stress-induced neural resource allocations in T+ individuals, shifting neural resources away from top-down control to salience detection, thereby likely rendering them more vulnerable to detrimental stress effects.

Alternatively, it is possible that these group differences represent markers of resilience in T+ individuals. We thoroughly excluded participants with a prior or current psychiatric disorder and did not find behavioural impairments, which indicates a relatively high level of resilience in this group. However, the current T+ group showed subtle psychiatric alterations on the behavioural level (i.e. higher depressive symptoms, higher trait anxiety, more posttraumatic stress symptoms). Importantly, a blunted IFG signal under stress was related to more posttraumatic symptom severity as well as higher anxiety values. This association further suggests that the observed alteration in the IFG signal in T+ participants is indicative of increased vulnerability rather than resilience. In line with this, both, hypoactivity of the IFG as well as hyperactivity of the right anterior insula has been implicated in the development of trauma-associated psychiatric diseases (e.g. Rauch *et al*., 1996; Stein *et al*., 2007; Hayes *et al*., 2012). In contrast, studies on neural markers of resilience point to increased PFC activation during top-down control (New *et al*., 2009; Blair *et al*., 2013), lower limbic activation during emotionally evocative events (Britton *et al*., 2005) and higher reactivity of the reward system (Vythilingam *et al*., 2009). Although the period of greatest risk of developing a psychiatric disorder has passed considering the mean age of T+ participants, a subgroup may still develop a mental disorder over time, depending on the magnitude of environmental stressors. Future studies should employ a longitudinal approach to consider neural differences between those that do and those that do not.

Functional differences of the IFG only arose under stress but did not appear at baseline. In contrast, previous studies in PTSD and other trauma-related disorders have consistently found decreased IFG activity at baseline across tasks. In their meta-analysis, Hayes *et al*. (2012) highlighted the IFG as a promising target for evaluating therapeutic success in PTSD. The lack of baseline differences in our study indicates that trauma *per se* might not be associated with baseline IFG reactivity, but that baseline IFG activity changes only occur after the development of PTSD. Our findings therefore support the idea of a gradient in terms of durable effects of trauma upon exposed individuals.

We found an increased anterior insula response to stress in T+ participants. This finding needs to be interpreted with caution because neural activation differences did not survive our very conservative *a-priori* threshold (*p* < 0.05 whole brain pTFCE and peak-level FDR correction). However, using a more liberal threshold, we found a large cluster of activation (*p* < 0.001 uncorrected, *k* = 40 voxels). Underlining the relevance of the observed neural activation for the behavioural process in T+ individuals, anterior insula activation was related to FARs on fearful faces. Moreover, heightened insula activation is in accordance with numerous reports of trauma-induced insula sensitization and an overactive salience network in trauma-related disorders (Patel *et al*., 2012; Ruocco *et al*., 2013; Stark *et al*., 2015). Furthermore, our finding is plausible from a computational network perspective. This perspective suggests that the insula has three central characteristics: it represents the focal point of the salience network, it coordinates the interplay of different large-scale neural networks and it provides access to the motor system via strong coupling with the anterior cingulate cortex (Menon and Uddin, 2010). As executive and salience networks are believed to be competing for resources (Fox *et al*., 2009; Hermans *et al*., 2014; Homberg *et al*., 2017), heightened responsivity of the anterior insula might thus prevent adequate IFG-initiated regulation. Moreover, aberrant coordination by the anterior insula may cause impaired prefrontal network activation (Patel *et al*., 2012), potentially paving the way to typical trauma-related symptoms. Lastly, the positive correlation between stress-induced insula activation and false alarms on fearful faces in T+ participants may be due to increased coupling with the motor system.

In line with our hypothesis, we moreover observed group differences in brain–behaviour correlations. In the T− group, IFG activation under stress was related to better inhibition of fearful faces under stress, which seems to be disrupted in T+ individuals. Moreover, in T+ only, stress-associated anterior insula activation positively correlated with stress-associated false alarms on fearful stimuli. Contrary to our expectation, we did not find a compensatory mechanism that preserves neural facilitation of emotion inhibition under stress in trauma-exposed individuals. As previous studies have demonstrated alterations in neural connectivity in trauma-exposed individuals (Brown *et al*., 2013; Cisler *et al*., 2013), future studies may examine potential compensatory mechanisms at the brain network level.

Furthermore, stress effects on emotion inhibition were limited to fearful stimuli. Previous investigations have been inconsistent with respect to the valence specificity of stress effects (e.g. Li *et al*., 2014; van Leeuwen *et al*., 2018). Here, relative levels of activation of sympathetic nervous system stimulation and the hypothalamic–pituitary–adrenal (HPA) axis might play a role. While exogenous cortisol (the end product of HPA axis activity) administration has mostly been found to lead to valence nonspecific effects, elevated noradrenergic signalling from the sympathetic nervous system may modulate stress-induced bias to negative cues, particularly in the presence of elevated cortisol levels (Kukolja *et al*., 2008). As stress induction in the present study activated noradrenergic signal increase as marked by *α*-amylase levels, combined with moderate HPA axis activation marked by cortisol levels, this could explain the observed specificity of stress effects on fearful stimuli inhibition. Fearful stimuli might be particularly difficult to regulate for T+ individuals under stress.

Finally, we would like to offer some additional considerations regarding our study. It is noteworthy that we carefully selected our experimental group of individuals exposed to severe and repeated early life trauma. Lifetime psychopathology and psychotropic medication were excluded by extensive diagnostic measures including structural clinical interviewing by a trained psychologist for every participant. While trauma exposure was assessed retrospectively, and thus susceptible to recall bias, unlike most related studies, we used the ETI (Bremner *et al*., 2000; Wingenfeld *et al*., 2011), a semi-structured interview to assess traumatic events in addition to questionnaires. Furthermore, we only recruited women to ensure homogeneity, but due to sex differences in acute HPA and autonomic reactivity (Kudielka and Kirschbaum, 2005), it is unclear whether results can be extrapolated to males. Due to a technical problem at the scanner during psychosocial stress induction, we were unable to record BOLD activation during the MIST in nine trauma participants and four controls. We therefore cannot draw reliable conclusions from this neural data during the MIST. Nonetheless, subjective stress ratings, the increase in heart rate and the endocrine data clearly show a pronounced stress response to the MIST. We provide details on MIST acquisition in the Supplementary materials and methods M7 and results in online Supplementary Tables S5 and S6.

It is important to note that we did not investigate acute trauma-exposure or exposure to trauma-trigger. The present study examined emotional response inhibition after an everyday life stressor, which is distinct from stress resulting from trauma or associated triggers. Moreover, early-life trauma-exposure was not associated with differences in ordinary stress-response when considering cortisol or *α*-amylase levels or reported stress.

Further, for power reasons, correct inhibition trials were used for the analysis of the BOLD signal underlying emotion inhibition. While the observed brain–behaviour correlations support the link between our chosen behavioural and neural substrates, the analysis of neural correlates of erroneous trials (misses as well as false alarms) would be of significant interest. Future studies might exclusively focus on fearful/neutral pictures to provide a sufficient number of erroneous trials. Moreover, replication in a larger sample is advisable in order to confirm results and potentially give insight into subtler (potentially compensatory) mechanisms at the brain network level. Lastly, participants were subjected to the eGNG task twice. Although we used parallel versions to minimize learning effects, we cannot exclude that learning effects might have influenced the results.

In summary, our data suggest that early life trauma has a durable effect on the brain, even in the absence of lifetime full-blown psychopathology. Dysregulation of top-down control by the IFG together with heightened bottom-up salience of fear related cues may alter neural stress processing and potentially increase vulnerability to adverse effects of everyday life stress.

## Supplementary material

For supplementary material accompanying this paper visit https://doi.org/10.1017/S0033291719002198. click here to view supplementary material
