# Supplementary material for: Healthy women with severe early life trauma show altered neural facilitation of emotion inhibition under acute stress
Source: Psychol Med. 2019 Aug 29;50(12):2075–84. doi: 10.1017/S0033291719002198 (PMC7525789; doi:10.1017/S0033291719002198)
Supplement: Supplementary file 1 [file S0033291719002198sup.zip › S0033291719002198sup001.docx]

# **Supplementary Information**

“Healthy women with severe early life trauma show altered neural facilitation of emotion inhibition under acute stress”

SUPPLEMENTARY MATERIALS AND METHODS

**M1) Order and timing of emotional go-nogo paradigm (eGNG)**

The naturalistic face stimuli used were drawn from the FACES database (Ebner et al., 2010), which are rated on mean accuracy (i.e. mean percentage of correct expression identification) and perceived age. Only faces of young and middle-aged actors with true as well as perceived (i.e. rated) ages of at least 18 and with highest possible accuracy ratings were selected. We chose to use fearful faces over angry faces as they have been found to be more perceptually salient and capable of holding attention (Engen et al., 2017). Pictures of the same actors, displaying the respective emotions, were used for all emotion conditions of one run. However, different sets of stimuli were used for each run (control vs. stress) to minimize learning effects. These sets were matched on mean age of actors and mean accuracy of facial expressions. The order of the blocks (i.e. the emotion condition) was counterbalanced for each participant but kept the same within participants for both runs. Stimuli were presented pseudo-randomly for 500 ms each and separated by a jittered inter-stimulus interval of 2000-10000 ms, during which participants viewed a blank screen. The jitter distribution was separated in steps of 250 ms and had an exponential slope with fewer long jitter times. The participants response was recorded until the stimulus disappeared after 500 ms. Presentation of the stimulus picture was taken as onset for fMRI trials, the duration of trials was 500 ms. One whole block lasted at most 2:57 minutes.

**M2) Psychosocial stress induction by an adapted version of the Montreal Imaging Stress Task (Dedovic *et al.*, 2005)**

The control condition consisted of easy arithmetic questions for which sufficient time was allotted (7 min). The stress condition included two runs (8 min each) with difficult arithmetic questions under time pressure. Here, time constraints and difficulty were automatically adapted to the subjects’ performance to result in failure rates of 50-55%, and a performance bar depicted the participants’ cumulative performance progressively falling behind the performance of a fake comparison group regardless of actual performance. After the first and before the second stress run, a confederate, introduced to the participant as the head of the study, delivered scripted negative feedback over the microphone emphasizing the need to subsequently improve performance for ̴1min.

**M3) Peak detection of pulse oximetry data (heart rate)**

TAPAS PhysIO toolbox, version r671, implemented in SPM12 (Kasper *et al.*, 2017) was used for processing of pulse oximetry data. Automated peak detection was performed by the adaptive ‘auto-matched’ peak detection algorithm of the PhysIO toolbox. Afterwards, diagnostic plots of the toolbox were used to manually check for outliers and wrongly detected heart beats due to movement and MR-signal related artefacts. All data sets with more than three wrongly detected heart beats (as indicated by the toolbox) were excluded from the analyses. Mean heart rate for presentation of arithmetic questions (Montreal Imaging Stress Task) and eGNG under control and stress condition was calculated.

**M4) Storage and biochemical analysis of salivary cortisol and alpha-amylase**

Salivette sampling device (Salivettes®, blue cap; Sarstedt Inc.) was used to collect samples. Collection took place at room temperature, after which samples were stored at -20°C until the end of the testing session and subsequently kept at -80°C until biochemical analysis. Salivary free cortisol and α-amylase levels were determined by the Neurobiology Laboratory of the Department of Psychiatry, Charité – Universitätsmedizin Berlin, Campus Benjamin Franklin. After thawing, Salivettes® were centrifuged for 2 minutes at 1000 x g.

Free cortisol was analysed using an adapted homogenous time-resolved fluorescence resonance energy transfer (HTR-FRET)-based competitive immunoassay, which is based on an anti-cortisol antibody labelled with Europium 3+-cryptate as the donor dye, and authentic cortisol conjugated with a second generation acceptor dye (D2) (Cisbio International, Codolet, France). In brief, 2 parts of the sample were subjected to a fluorescence microtiter plate and 1 part of D2-conjugated cortisol was added immediately thereafter. Both components (saliva and D2-conjugate) were thoroughly mixed using a multi-channel pipette and centrifuged for 2 minutes at 1000 x g using a microtiter plate centrifuge (Heraeus Biofuge, Thermo Fisher Scientific, Braunschweig, Germany). After centrifugation, 1 part of Eu3+-cryptate-labelled anti-cortisol antibody was added, again thoroughly mixed, centrifuged (2 minutes at 1000 x g) and allowed to incubate for at least 2h. Appropriate authentic standards, negative, positive and blank controls were included according to the manufacturer’s instructions. After incubation, time-resolved fluorescence was measured at 620 nm and 665 nm using a Clariostar multimode plate reader (BMG Labtech, Ortenberg, Germany). Increase in fluorescence at 665 nm (acceptor fluorescence of D2) was normalized to fluorescence at 620 nm (donor fluorescence of Eu3+-cryptate) to account for differences in plating volumes or micro bubbles, and calculated as relative increase in fluorescence over Eu3+-cryptate-only containing blanks. Intraassay coefficients of variation were below 8%, interassay coefficients of variation were below 10 %. All samples and standards were measured in duplicates. The limit of detection of free cortisol was 0.2 nM. Intra-assay coefficients of variation were below 8%, inter-assay coefficients of variation were below 10% for both cortisol and α-amylase. All samples and standards were measured in duplicates (also see Duesenberg *et al.*, 2016).

Alpha-amylase activity was determined using a modified protocol of a previously published direct alpha-amylase assay (Rombold *et al.*, 2016). In brief, saliva samples were assessed in triplicates using 2-chloro-4-nitro-phenyl-a-D-maltotrioside (CNPG) as a chromogenic substrate, kinetic measurements were performed by measuring changes in optical density at 405 nm after 2, 4, 6 and 8 minutes.

In detail, alpha-amylase activity was determined using a modified protocol of a previously published direct alpha-amylase assay (Lorentz et al., Clin Chem Lab Med 1999; 37(11/12):1053–1062). The assay principle follows an IFCC method using 2-chloro-4-nitro-phenyl-a-D-maltotrioside (CNPG) as the chromogenic substrate and was adapted to be run at room temperature using six authentic alpha-amylase standards (Sigma-Aldrich, Taufkirchen, Germany) for absolute quantification. In brief, samples and standards were diluted 1:200 and 1 part of sample/standard was subjected to a clear microtiter plate. Plates were subsequently allowed to equilibrate to 20 °C inside the temperature-controlled multimode plate-reader and 20 parts of CNPG-containing chromogenic substrate (substrate start procedure) were injected in a time-controlled manner. Following each automated injection, the plate was shaken and an increase in absorbance was read at 405 nm periodically after exactly 2, 4, 6 and 8 minutes in each well. A linear increase in absorbance was assured for all samples and average increase in absorbance per minute (delta OD405 / min) was calculated for all samples and standards. Absolute quantification of alpha-amylase activity in samples was realized by 4-parameter nonlinear regression analysis of the calibration standards (r2 > 0,998). Inter- and intraassay coefficients of variation were both lower than 10 % for alpha-amylase activity. All samples and standards were measured in triplicates.

**M5) fMRI acquisition parameters and pre-processing of the emotional go-nogo paradigm**

FMRI data were collected with a 3 Tesla Magnetom Trio scanner system (Siemens Medical Systems, Erlangen, Germany) using a 12-channel radiofrequency head coil. Stimuli were projected onto a screen at the end of the magnet bore by a video projector (NEC GT950 NEC Corporation, Itasca, IL, USA, resolution 1024×768 pixels), which participants viewed via a mirror on the head coil. Participants responded by pushing a fiber-optic light sensitive key press. Functional images were collected using a standard echo planar imaging (EPI) sequence with 37 axial slices of 3 x 3 mm (interleaved ascending order, field of view (FoV) 192 mm, repetition time (TR) 2 s, echo time (TE) 30 ms, flip angle 70°, matrix size = 64 x 64). Two sessions, each with 4 runs of 94 volumes were acquired. For anatomical reference, a T1-weighted high-resolution magnetization prepared gradient-echo scan (MPRAGE) was obtained (176 slices, TR = 1900, flip angle 9°, TE = 2.52 ms, FoV = 256 mm, matrix size = 256 x 256, voxel size = 1 x 1 x 1 mm^3^).

FMRI data were analysed using MATLAB R2014a (The Mathworks Inc., Natick, MA, USA) and SPM12 (Statistical parametric mapping software, SPM; Wellcome Department of Imaging Neuroscience, London, UK). First, EPI-images were slice-timing corrected and realigned to the first volume, thereby correcting for head movement (six-parameter rigid body transformation). The anatomical MPRAGE was co-registered to the mean EPI and segmented. The acquired segmentation matrix was further used for the normalization of functional (voxel size = 3 x 3 x 3 mm^3^) and structural images (voxel size = 1 x 1 x 1 mm^3^) to the stereotactic normalized standard space of the Montreal Imaging Institute. In a last step, data were spatially smoothed utilizing an 8 mm (full width at half maximum) Gaussian kernel. The time series was high-pass filtered to eliminate low-frequency components (filter width 128 s).

**M6) Supplementary analysis assessing neural response during the emotional go-nogo paradigm when controlling for cortisol levels**

As a supplementary analysis, we added cortisol level increases during stress induction (∆T2 to T3) as a covariate into the flexible factorial ANOVA. The model thus contained the main factors group, emotion condition, subject as well as an interaction term cortisol x group. The same thresholding approach as in the main analysis was used (probabilistic threshold-free cluster enhancement and whole brain peak level FDR-correction with p < 0.05 and a minimum cluster size of k > 30).

Results for the principal controls comparing fearful faces (fearful nogo) to neutral nogo ones and testing for group differences were similar. The difference in left IFG activation between the groups became slightly more pronounced (MNI: -42, 38, -4, t = 5.77, p = 0.003 pTFCE and peak-level FDR-corrected, 941 voxels) whereas the group difference in right anterior insula activation was slightly smaller (MNI: 33, 14, 11, t = 3.51, p < 0.001 uncorrected, 24 voxels).

**M7) Analysis of neural correlates during stress induction (Montreal Imaging Stress Task)**

Due to a technical problem at the scanner in 13 cases during the Montreal Imaging Stress Task, the final sample for the analysis of neural correlates during this paradigm was restricted to 14 trauma participants and 19 trauma-naïve control participants for control and stress run 2, and 18 control participants (14 trauma) for stress run 1. Therefore, these data are not reported or discussed in the main text, but for completeness, results are reported in Table S6.

Acquisition parameters

Three EPI sequences were acquired, the first (control) consisting of 220 volumes and the other two (stress) sessions of 250 volumes.

Acquisition parameters for the EPIs were identical with those reported for the EPI sequences of the eGNG. Also, the same T1-weighted MPRAGE (176 slices) was used for anatomical reference.

Imaging analysis of stress induction

Effects were estimated with a general linear model (GLM) convolving each block with a hemodynamic response function. The time-series was high-pass filtered with a cut-off frequency of 300 s. A fixed-effect model was performed to create images of parameter estimates. The model contained three regressors (control, stress 1, stress 2) as well as six motion regressors of no interest. Individual *t*-maps of contrasts [stress 2 > control] as well as [stress 2 vs. stress 1] were computed and taken to group level.

At the group level, these individual *t-*maps were entered into two one-sample *t*-tests across all participants to examine neural correlates of stress. Subsequently, two two-sample *t*-test were computed (using the same *t*-maps) to evaluate group differences (T+ vs. T-).

**M8) one-way ANCOVA to analyse group differences in stress-associated left IFG activation when controlling for posttraumatic symptoms and trait anxiety**

With this one-way analysis of covariance (run in SPSS) we followed up on observed significant negative correlations between left stress-induced IFG activation and both PDS total score and STAI-trait. Group was entered as a between subject factor, PDS total score and STAI-trait scores as covariates, and extracted parameter estimates for stress-associated left IFG activation as a dependent variable. The assumptions for an ANCOVA were met. Results showed a significant effect of group even when controlling for posttraumatic symptoms and trait anxiety (F_1,42_ = 6.278, p = 0.016).

SUPPLEMENTARY TABLES

**Table S1:** Regressors for emotion condition fearful nogo – neutral go (for illustrative purposes) in first level model

| **Regressors** | **Trials** |
| --- | --- |
| Regressor 1 | All trials presented in control condition where participant correctly pressed a button when a neutral face was displayed (correct go). |
| Regressor 2 | All trials presented in control condition where participant correctly refrained from pressing a button when a fearful face was displayed (correct nogo). |
| Regressor 3 | All trials presented in control condition where participant failed to press to press a button when a neutral face was displayed (misses). |
| Regressor 4 | All trials presented in control condition where participant incorrectly pressed a button when a fearful face was displayed (false alarms). |
| Regressor 5 | All trials presented in stress condition where participant correctly pressed a button when a neutral face was displayed (correct go). |
| Regressor 6 | All trials presented in stress condition where participant correctly refrained from pressing a button when a fearful face was displayed (correct nogo). |
| Regressor 7 | All trials presented in stress condition where participant failed to press to press a button when a neutral face was displayed (misses). |
| Regressor 8 | All trials presented in stress condition where participant incorrectly pressed a button when a fearful face was displayed (false alarms). |

Note: Regressors were equivalent for the other three emotion conditions.

**Table S2:** Means and standard deviation for heart rate measurement during control and stress condition for both groups

| **Heart Rate** | | | |
| --- | --- | --- | --- |
|  | | **T-** (N=18)  Mean (SD) | **T+** (N=20)  Mean (SD) |
| **Control** | Math | 74.02 (10.20) | 70.23 (9.34) |
|  | eGNG | 67.95 (9.19) | 66.45 (9.31) |
| **Stress** | Math | 76.86 (8.27) | 72.00 (9.69) |
|  | eGNG | 71.74 (11.29) | 68.64 (9.09) |

*Note:* 3 T+ data sets and 5 T- had to be excluded due to movement and scanner related artefacts (N_T-_ = 18, N_T+_ = 20); *Abbreviations:* eGNG = emotional go-nogo task, T+ = trauma-exposed participants, T- = trauma-naïve participants, SD = standard deviation

**Table S3:** Means and standard error for cortisol and α-amylase for all time points

| **Cortisol** | | | | |
| --- | --- | --- | --- | --- |
| **Time Point** | **Raw**  Mean (SEM) | | **LN**  Mean (SEM) | |
|  | **T-** | **T+** | **T-** | **T+** |
| T1 | 1.43 (0.13) | 1.20 (0.22) | 0.25 (0.11) | -0.08 (0.15) |
| T2 | 1.18 (0.17) | 1.17 (0.37) | -0.07 (0.15) | -0.44 (0.19) |
| T3 | 1.40 (0.26) | 1.34 (0.24) | 0.06 (0.16) | -0.04 (0.19) |
| T4 | 1.14 (0.22) | 1.40 (0.43) | -0.20 (0.18) | -0.29 (0.20) |
| T5 | 0.99 (0.19) | 1.34 (0.36) | -0.30 (0.16) | -0.24 (0.19) |
| **α-Amylase** | | | | |
| **Time Point** | **Raw**  Mean (SEM) | | **LN**  Mean (SEM) | |
|  | **T-** | **T+** | **T-** | **T+** |
| T1 | 100.17 (13.57) | 136.35 (14.00) | 4.36 (0.18) | 4.78 (0.12) |
| T2 | 114.13 (19.57) | 117.24 (18.78) | 4.41 (0.19) | 4.51 (0.15) |
| T3 | 172.36 (25.13) | 189.07 (24.77) | 4.83 (0.20) | 5.03 (0.15) |
| T4 | 130.86 (18.67) | 151.67 (21.03) | 4.60 (0.18) | 4.79 (0.15) |
| T5 | 126.34 (19.17) | 132.72 (19.36) | 4.57 (0.18) | 4.63 (0.16) |

*Note:* One T- participant had to be excluded (N_T-_= 22, N_T+_ = 23); *Abbreviations:* LN = natural logarithm, T+ = trauma-exposed participants, T- = trauma-naïve participants, SEM = standard error of the mean

**Table S4:** Statistics of stress effects on emotional response inhibition (eGNG)

| **Hits** | | | | | |
| --- | --- | --- | --- | --- | --- |
| **Stimulus** | | **Control**  Mean (SEM) | **Stress**  Mean (SEM) | **WSR** | **Effect Size** (Pearson) |
| Fearful go | | 27.76 (.538) | 29.54 (.155) | Z=-4.08, *p* < 0.001 | *r* = 0.60 |
| Happy go | | 29.67 (.094) | 29.98 (.022) | Z=-3.13, *p* < 0.001 | *r* = 0.46 |
| Neutral go (Fearful nogo) | | 28.76 (.214) | 29.65 (.104) | Z=-3.96, *p* < 0.001 | *r* = 0.58 |
| Neutral go (Happy nogo) | | 28.43 (.433) | 29.63 (.133) | Z=-3.46, *p* < 0.001 | *r* = 0.51 |
| **Reaction Time on Hits** | | | | | |
| **Stimulus** | **Control**  Mean (SEM) | | **Stress**  Mean (SEM) | **WSR** | **Effect Size** (Pearson) |
| Fearful go | 0.50 (.012) | | 0.47 (.019) | Z=-3.49, *p* < 0.001 | *r* = 0.51 |
| Happy go | 0.46 (.010) | | 0.45 (.010) | Z= 1.34, *p* = 0.183 | *r* = 0.20 |
| Neutral go (Fearful nogo) | 0.50 (.011) | | 0.49 (.018) | Z=-3.91, *p* < 0.001 | *r* = 0.58 |
| Neutral go (Happy nogo) | 0.51 (.012) | | 0.49 (.015) | Z=-3.55, *p* < 0.001 | *r* = 0.52 |

*Note.* SEM = standard error; WSR = Wilcoxon signed-rank test

**Table S5**: Comparison between control and high stress run with one-sample *t*-test (N=33)

| **Region** | **H** | **Cluster size**  (voxels) | **t**  (peak) | **p**  (FDR cluster level) | **MNI coordinates**  (peak) | | |
| --- | --- | --- | --- | --- | --- | --- | --- |
|  |  |  |  |  | x | y | z |
| **t-contrast: Stress 2 > control** | | | | | | | |
| Middle Temporal | R | 247 | 7.15 | 0.001 | 42 | -64 | 5 |
| Superior Occipital | R | 257 | 5.87 | 0.001 | 24 | -88 | 26 |
| Middle Occipital | L | 192 | 5.08 | 0.002 | -36 | -70 | 11 |
| Precuneus | R | 99 | 4.71 | 0.029 | 12 | -43 | 56 |
| Occipital Pole | L | 82 | 5.40 | 0.042 | -18 | -100 | 14 |
| **t-contrast: control > Stress 2** | | | | | | | |
| Posterior-Medial Frontal | L | 864 | 6.52 | <0.001 | -9 | 23 | 65 |
| Middle Temporal | L | 223 | 6.19 | 0.002 | -48 | -34 | -4 |
| Angular | L | 119 | 5.75 | 0.025 | -45 | -67 | 47 |
| Inferior Temporal | R | 102 | 5.45 | 0.033 | 48 | 2 | -34 |

*Note.* Abbreviations: H = hemisphere, R = right, L = left

**Table S6:** Comparison between high stress (stress 2) and moderate stress (stress 1) run with one-sample *t*-test (N=32)

| **Region** | **H** | **Cluster size**  (voxels) | **t**  (peak) | **p**  (FDR cluster level) | **MNI coordinates**  (peak) | | |
| --- | --- | --- | --- | --- | --- | --- | --- |
|  |  |  |  |  | x | y | z |
| **t-contrast: Stress 2 > Stress 1** | | | | | | | |
| Calcarine / Precuneus / Lateral Occipital | L | 64 | 6.00 | 0.044 | -30 | -55 | 14 |
| **t-contrast: Stress 1 > Stress 2** | | | | | | | |
| Middle Temporal / Angular | R | 706 | 6.09 | <0.001 | 51 | -4 | -25 |
| Middle/ Superior Temporal | L | 423 | 6.39 | <0.001 | -60 | -43 | 11 |
| Calcarine / fusiform | bil | 350 | 5.52 | <0.001 | 0 | -97 | -1 |
| Precuneus / Posterior Cingulate | R | 289 | 4.75 | <0.001 | 3 | -55 | 26 |
| Superior Frontal | R | 282 | 6.05 | <0.001 | 15 | 65 | 23 |
| Posterior-Medial Frontal | R | 170 | 7.34 | <0.001 | 9 | 23 | 68 |
| Superior Occipital | R | 142 | 5.19 | 0.003 | 21 | -91 | 32 |
| Hippocampus / Parahippocampal | L | 82 | 6.60 | 0.026 | -24 | -25 | -22 |
| Middle Frontal | R | 81 | 4.47 | 0.026 | 45 | 14 | 53 |
| Superior Frontal | L | 80 | 6.15 | 0.026 | -24 | 59 | 26 |
| Cerebellum | L | 66 | 5.02 | 0.045 | -27 | -85 | -25 |

*Note.* Group comparisons (T+ vs. T-) by means of two-sample t-tests did not yield any suprathreshold clusters. Abbreviations: H = hemisphere, R = right, L = left, bil = bilateral

SUPPLEMENTARY FIGURES


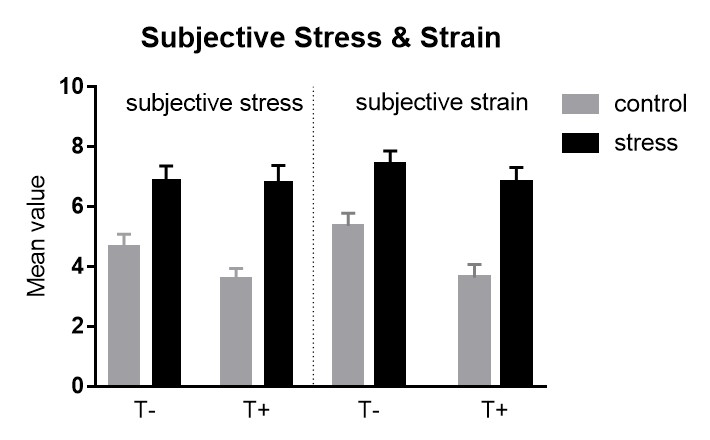


*Figure S1*. Subjective stress and strain during control versus stress condition in both groups. A mixed ANOVA with within-subject factor subjective stress (control vs. stress condition) and between subject factor group showed a significant main effect of subjective stress (N = 44, F_1,42_ = 88.4, *p* < 0.001) with higher subjective stress in the stress compared to the control condition across groups (M_control_ = 4.1 ± 2.0, M_stress_ = 6.9 ± 2.3), no main effect of group (*p* = 0.346) and a trend for a group by stress interaction (*p* = 0.097). In an equivalent mixed ANOVA of subjective strain, there was a main effect of strain (N = 44, F_1,42_ = 92.7, *p* < 0.001), a marginally significant group effect (F_1,42_ = 3.9, *p* = 0.056) and a significant group by strain interaction (F_1,42_ = 7.1, *p* = 0.042). Thus, subjective strain increased in both groups. Since in the control condition, subjective strain was slightly lower for the T+ group compared to the T- group, the increase from control to stress condition was slightly larger in the T+ group. Error bars represent standard errors.


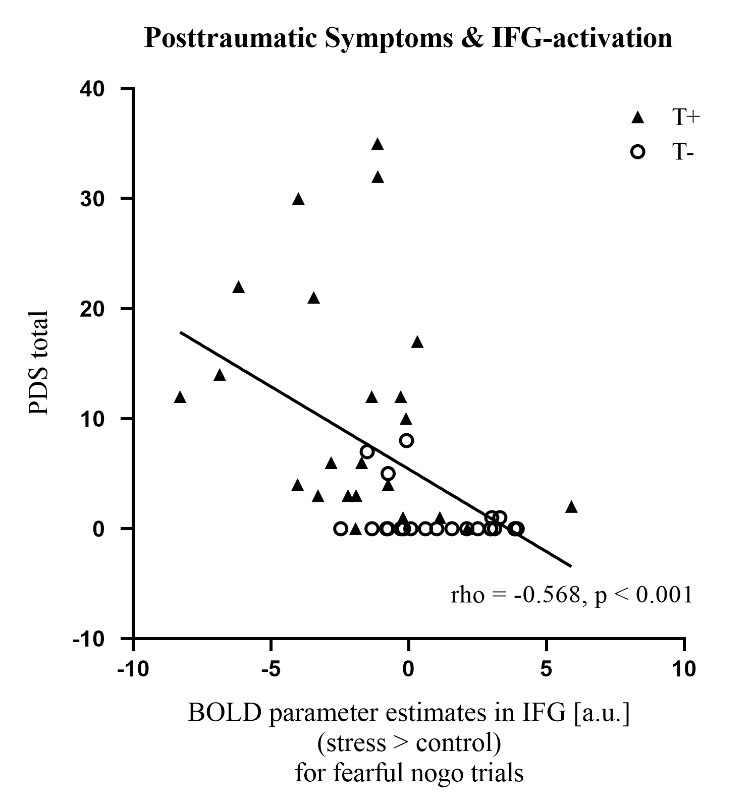


*Figure S2.* Significant negative Spearman correlation between posttraumatic symptoms during the last month (PDS total) and stress induced left IFG activation during fearful nogo-trials [i.e. stress > control]. *Abbreviations:* T- = trauma-naïve control participants, T+ = trauma-exposed participants, PDS = posttraumatic diagnostic scale, IFG = inferior frontal gyrus

References

**Dedovic, K., Renwick, R., Mahani, N. K., Engert, V., Lupien, S. J. & Pruessner, J. C.** (2005). The Montreal Imaging Stress Task: using functional imaging to investigate the effects of perceiving and processing psychosocial stress in the human brain. *Journal of Psychiatry and Neuroscience* **30**, 319-325.

**Duesenberg, M., Weber, J., Schulze, L., Schaeuffele, C., Roepke, S., Hellmann-Regen, J., Otte, C. & Wingenfeld, K.** (2016). Does cortisol modulate emotion recognition and empathy? *Psychoneuroendocrinology* **66**, 221-227.

**Ebner, N. C., Riediger, M. & Lindenberger, U.** (2010). FACES—A database of facial expressions in young, middle-aged, and older women and men: Development and validation. *Behavior Research Methods* **42**, 351-362.

**Engen, H. G., Smallwood, J. & Singer, T.** (2017). Differential impact of emotional task relevance on three indices of prioritised processing for fearful and angry facial expressions. *Cognition and Emotion* **31**, 175-184.

**Kasper, L., Bollmann, S., Diaconescu, A. O., Hutton, C., Heinzle, J., Iglesias, S., Hauser, T. U., Sebold, M., Manjaly, Z.-M., Pruessmann, K. P. & Stephan, K. E.** (2017). The PhysIO Toolbox for Modeling Physiological Noise in fMRI Data. *Journal of Neuroscience Methods* **276**, 56-72.

**Rombold, F., Wingenfeld, K., Renneberg, B., Hellmann-Regen, J., Otte, C. & Roepke, S.** (2016). Influence of the noradrenergic system on the formation of intrusive memories in women: an experimental approach with a trauma film paradigm. *Psychological Medicine* **46**, 2523-2534.
